# Spontaneous rupture of a normal spleen successfully treated with elective laparoscopic splenectomy: a case report

**DOI:** 10.1093/jscr/rjag178

**Published:** 2026-03-21

**Authors:** Ryoma Sakamoto, Yuto Kawate, Kazuki Sekine, Ken Hayashi, Akinari Miyazaki

**Affiliations:** Department of Gastrointestinal Surgery, Kameda Medical Center, 929, Higashimachi, Kamogawa City, Chiba 2960041, Japan; Department of Pediatric Surgery and Transplantation, Kumamoto University Hospital, 1-1-1 Honjo, Chuo Ward, Kumamoto City, Kumamoto 8608556, Japan; Department of Hepatobiliary Pancreatic Surgery, Juntendo University Hospital, 2-1-1 Hongo, Bunkyo-ku, Tokyo 1138421, Japan; Department of Gastrointestinal Surgery, Kameda Medical Center, 929, Higashimachi, Kamogawa City, Chiba 2960041, Japan; Department of Gastrointestinal Surgery, Kameda Medical Center, 929, Higashimachi, Kamogawa City, Chiba 2960041, Japan

**Keywords:** spontaneous rupture, spleen, non-operative management, laparoscopic splenectomy

## Abstract

Spontaneous splenic rupture is a rare but potentially life-threatening condition. Although commonly associated with infections or malignancies, rupture of a normal spleen is extremely rare, and emergency surgery remains the usual management approach. We report a case of spontaneous rupture of a normal spleen successfully treated with elective laparoscopic splenectomy. A 47-year-old man presented with sudden-onset left upper-quadrant abdominal pain and dizziness. Imaging studies revealed splenic rupture without any evidence of trauma or underlying conditions. After initial non-operative management, laparoscopic splenectomy was performed due to the risk of rebleeding. Pathological examination of the excised spleen revealed no underlying cause, and the patient was diagnosed with spontaneous rupture of a normal spleen. A PubMed-based literature review identified only a few comparable cases over the past 5 years, most treated with emergency surgery. Our case indicates that non-operative management followed by elective surgery may be feasible in selected cases.

## Introduction

Spontaneous splenic rupture (SSR) typically occurs secondary to trauma, infection, hematological disease, or malignancy. However, rupture of histologically normal spleens without any apparent cause, termed “spontaneous rupture of a normal spleen”, is extremely rare. We present a case of SSR in a patient with no identifiable risk factors, who was initially managed conservatively, and later underwent elective laparoscopic splenectomy. A brief literature review is included to highlight this unique clinical presentation.

## Case report

**Figure 1:**
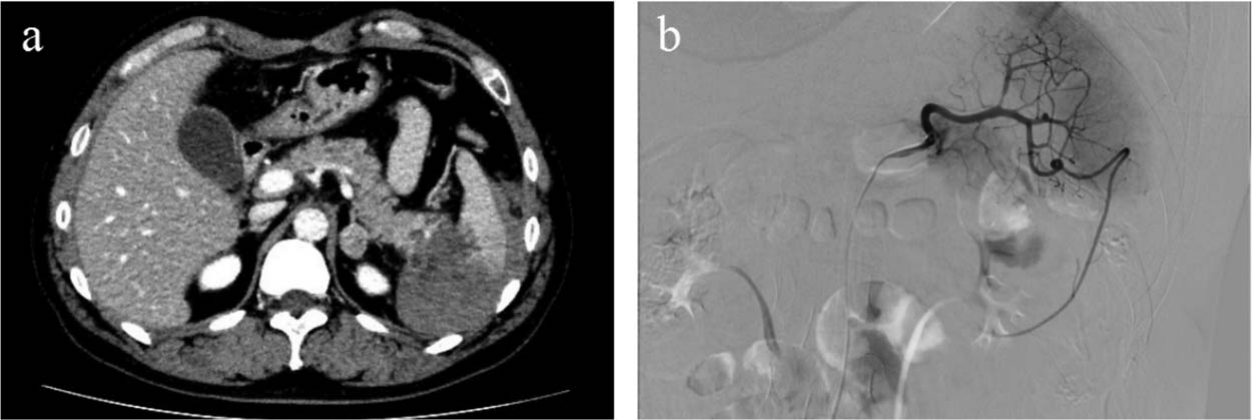
Hemorrhagic ascites were observed primarily around the spleen, suggesting splenic rupture. No contrast extravasation was observed and active bleeding was not suspected (a). Angiography showed no active bleeding or splenic artery aneurysms were observed on angiography (b).

**Figure 2:**
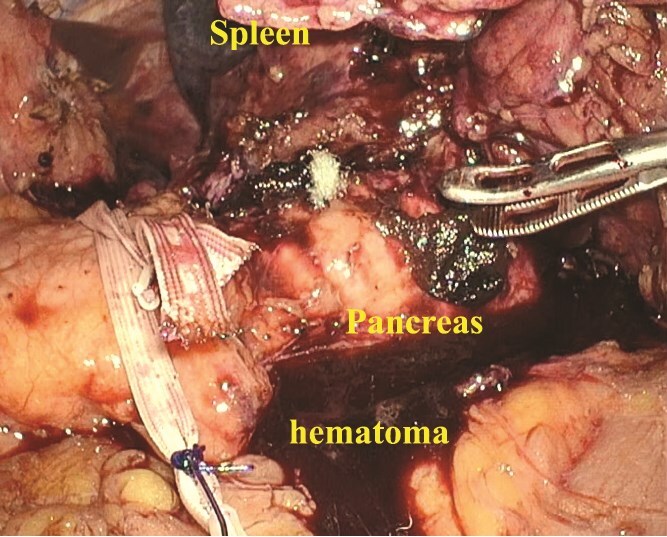
A large amount of hematoma was detected within the abdominal cavity. Owing to the hematoma, dense adhesions were formed between the spleen and surrounding tissues.

A 47-year-old male presented to our emergency department with sudden onset of left upper-quadrant (LUQ) abdominal pain that began 3 days earlier and an episode of dizziness that had happened on the day of presentation. His medical history included gout and benign prostatic hyperplasia, for which he was regularly prescribed febuxostat. There was no history of anticoagulant use. He denied any history of alcohol or illegal drug use. There was no history of taking nutritional supplements prior to his initial visit. There was no family history of similar events or hematological disorders. The patient had no history of recent trauma or involvement in contact sports. He was engaged in the ferry business and worked as a ferryman. On arrival, his vital signs were stable with a blood pressure of 135/88 mmHg, heart rate of 97 bpm, body temperature of 35.6°C, and oxygen saturation of 100% on room air. Physical examination revealed mild tenderness in the LUQ without rebound tenderness. Laboratory examination showed leukocytosis with a white blood cell count of 16 600/μl and an elevated C-reactive protein level of 4.11 mg/dl. The hemoglobin level was 15.5 g/dl, showing no decrease. Both prothrombin time and activated partial thromboplastin time were within normal limits (prothrombin time-international normalized ratio: 0.89, activated partial thromboplastin time: 35.2 s). Platelet count was also within the normal range (231 000/μl). There were no increases in amylase or lipase level (amylase: 44 U/l, lipase: 8 U/l). Contrast-enhanced computed tomography (CT) revealed hemorrhagic ascites predominantly surrounding the spleen, suggestive of splenic rupture, although no active bleeding or splenic masses were observed (Fig. 1a). The size of the spleen was within normal limits (92 × 49 mm). There were no available previous images because this was the initial visit of the patient. CT revealed advanced hepatosteatosis, but no other morphological abnormalities suggestive of liver disease were observed. A follow-up CT scan 5 h after the initial scan showed only a slight increase in hemorrhagic ascites with no signs of active hemorrhage. Angiography performed the same day showed no active bleeding or aneurysm formation in the splenic artery (Fig. 1b). Given that the patient remained hemodynamically stable and no decline in hemoglobin level was observed, non-operative management was initially selected. Magnetic resonance imaging and endoscopic ultrasonography were performed to identify the cause of the splenic rupture; however, they failed to detect any underlying conditions. Although we could not determine the cause of the rupture, the patient was stable after admission and was discharged on the seventh hospital day. Given the persistent risk, laparoscopic splenectomy was performed 24 days after the initial presentation. A large hematoma is observed around the spleen (Fig. 2). Owing to the hemorrhagic event, dense adhesions were formed between the spleen and surrounding tissues, particularly the diaphragm. The adhesion between the pancreatic tail and spleen was strong, making it challenging to separate them. The pancreatic tail was resected along the spleen. Other than biochemical pancreatic leak, no post-operative events occurred, and the patient was discharged on post-operative Day 12. The resected spleen was histologically normal and no causal lesions were noted. A hematology consultation was obtained to evaluate the possibility of an underlying hematologic disorder; however, lymphoma, leukemia, myeloproliferative neoplasms, and other hematologic disorders that could potentially cause splenic rupture were ruled out based on laboratory findings, imaging studies, and histopathological examination. We discussed the patient’s condition with the department of pathology, and concluded that spontaneous rupture of a normal spleen was the most likely diagnosis.

## Discussion

Spontaneous rupture of a normal spleen is extremely rare because most splenic ruptures occur secondary to malignancy, infection, or trauma. When this disease concept was first proposed [1], the definition of spontaneous rupture became ambiguous. Orloff *et al.* conducted a comprehensive literature review and established the following four diagnostic criteria [2]:

- No history of trauma or unusual effect which could injure the spleen.
- No evidence of disease in other organs which could adversely affect the spleen.
- No evidence of peri-splenic adhesions or scarring of the spleen which suggest that it had been traumatized or had ruptured previously.
- Splenic normality on macroscopic and microscopic examinations

Crate *et al.* later suggested adding a fifth criterion [3]: the absence of laboratory evidence of viral infection that could affect the spleen.

The patient had a medical history of gout and hepatosteatosis and was on regular febuxostat therapy, but there are no reports suggesting that these conditions or this medication are associated with splenic rupture. Our case fulfilled all these criteria and was diagnosed as a spontaneous rupture of a normal spleen. We identified 96 case reports containing the term “spontaneous splenic rupture” archived in PubMed over the past 5 years (2020–2024). Of these, only five cases met the diagnostic criteria for spontaneous rupture of a normal spleen [4–8]. The chief complaint was acute abdominal pain. No specific trends were observed regarding the age or sex of the patients. Emergency surgery was performed in nearly all cases; however, our case was the only one in which non-operative management succeeded and emergency surgery was avoided. In our case, the interval between symptom onset and initial presentation was longer than that observed in other cases. Moreover, unlike other cases, there was no decrease in blood pressure or hemoglobin levels. The rarity of spontaneous rupture of a normal spleen makes diagnosis and management challenging. This case highlights that in strictly selected hemodynamically stable patients, initial non-operative management followed by elective laparoscopic splenectomy may offer a safe and reasonable alternative to emergency surgery. Further accumulation of similar cases is essential to better understand this condition and improve its management.
